# Meteorological Register for January, 1817

**Published:** 1817-03

**Authors:** John Bailey

**Affiliations:** Surgeon, &c.


### Meteorological Register for January, 1817,

*Kept at Harwich, in Essex,*

By JOHN BAILEY, Surgeon, &c.

| | Moon's Age. | Atmospherical Preſſure. Morn. | Ev. | Temp. | Wind. | Remarks. | General Statement of Diſeaſes. |
|---|---|---|---|---|---|---|---|
| | 1 | 29 5 | 29 3 | 45 | W | R. Dull & foggy | |
| | 2 | 4 | 5 | 44 | SW | Rain. Fine | |
| ● | 3 | 4 | 6 | 36 | SW | ditto | Pneumonia |
| | 4 | 2 | 2 | 50 | SW | Rain.G.of wind | |
| | 5 | 7 | 5 | 40 | W | ditto. Dull | Rheumatismus |
| | 6 | 4 | 4 | 39 | W | do.cloudy&dull | |
| | 7 | 30 1 | 30 2½ | 32 | NW | Fine & clear | Catarrhus |
| | 8 | 2½ | 3 | 32 | W | ditto | |
| | 9 | 4 | 4 | 33 | Calm | ditto | |
| ☽ | 10 | 4 | 4 | 31 | W | Thick fog | |
| | 11 | 2½ | 1½ | 30 | NW | Foggy | |
| | 12 | ½ | 29 8½ | 32 | SW | Fog | |
| | 13 | 29 7 | 7 | 36 | SW | ditto | |
| | 14 | 4 | 4 | 36 | N | Cloudy | |
| | 15 | 28 9½ | 29 2 | 36 | SE | Snow | |
| | 16 | 29 3 | 28 8½ | 28 | SE | Rain, ditto | |
| ○ | 17 | 28 8½ | 8½ | 40 | SW | Cloudy | |
| | 18 | 29 1 | 29 1 | 43 | | Fine & clear | |
| | 19 | 1 | 28 8½ | 40 | SW | R. heavy show. | |
| | 20 | | | 40 | SW | ditto | |
| | 21 | 4½ | 8 | 37 | W | | |
| | 22 | 7 | 8 | 43 | W | Gale of wind | |
| | 23 | 8 | 8 | 48 | W | ditto | |
| | 24 | 30 ½ | 1½ | 50 | S | Foggy | |
| ☾ | 25 | 2 | 2 | 48 | W | ditto | |
| | 26 | 1 | 1 | 46 | S | ditto | |
| | 27 | 2 | 3 | 47 | E | Fine & clear | |
| | 28 | 3 | 1½ | 46 | | Foggy | |
| | 29 | 1½ | 1½ | 42 | NW | Fine & clear | |
| | 30 | 1½ | 1½ | 43 | NW | Foggy | |
| | 31 | 3 | 3 | 50 | N | Fine & clear | |

---

Wet weather in the Brumal quarter of the year, unless it be accompanied by a depression of the mercury in the thermometer, does not carry in its train a long list of atmospheric disease. The present month has not been an unhealthy one. Catarrhal affections have been general, but not violent. Acute Rheumatism and Pneumonia were the leading complaints.

